# Formula

**Published:** 1855-01

**Authors:** 


### FORMULA.

We suggest the following formula as worthy the consideration of the practitioner:

Syrup of Elderberry.—This is considered superior to the syrup Sarsaparilla, by those who have used it. The common elder shrub possesses in its root, bark, leaves and flowers, manifest medical properties; purgative, diaphoretic, diuretic, alterative, aperient, and the leaves a discutient as a poultice; and, it appears from recent trials, that the juice of the berries possesses alterative properties of a high order. This will be quite a desideratum to the practitioners of the West, as the shrub grows abundantly along the borders of our streams, and in the rich alluvial bottoms. It is the *sambucus canadensis* of Wood and Bache. To every pint of the expressed juice a pound of refined sugar is added, and then boiled to a syrup; and to every pint of syrup, an ounce (fluid) of good brandy is added, to prevent fermentation. Iodine, Iod. Pot., arsenic, &c., may be added, each as required.

There is no more medicinal property in Sarsaparilla, than in an equal quantity of *dry straw*, in our opinion.

---

### LIQUOR MORPHIA COMPOSITUS.

This is a beautiful preparation, and while we thank the Journal of Pharmacy for it, we will give it for the benefit of our readers, and can assure them after considerable experience in its use, that it is one or the very best anodyne preparations we have ever used, as it does not produce the cerebral symptoms to the same degree with any other forms of opium, as we have found it admissible when all the other forms had been rejected. In diarrhœa, and in dysentery, it has proved a most valuable anodyne, in children particlarly.

℞ Acetate morphia ℈j.,

Acetic acid dilute f ʒj.,

Dissolve and add—Dilute alcohol f ℥vij.,

Wine ipecac f ℥ij

M.

Ten drops equal to 1-8 grain morphia, and 1-8 grain ipecac, and 14 drops equal to one grain opium. In febrile diseases the wine ipecac might be increased to ℥iij with advantage. It is easily taken, and where accuracy is particularly required it can be attained, as one drop is the 14th part of a grain of opium. We have, however, given it in larger doses than the above.

